# Supplementary material for: A Likelihood Approach for Real-Time Calibration of Stochastic Compartmental Epidemic Models
Source: PLoS Comput Biol. 2017 Jan 17;13(1):e1005257. doi: 10.1371/journal.pcbi.1005257 (PMC5240920; doi:10.1371/journal.pcbi.1005257)
Supplement: S1 File — (TAR.GZ) [file pcbi.1005257.s014.tar.gz › HSPH_Online-SI-Revision/MSS10/n1-severe/n1-severe_NEW_table-4.pdf]

1	R0	{1.51081, 13.7078, 1.}	{1.16074, 2.63384, 0.07}	{{1.06738, 2.19254, 0.}, {1.25474, 3.26445, 0.}}	{23.171, 80.7858, 93.}	inf	45.	16.3075	{3.86884, 28.7718}	64.0273	0.539338	newly	25.	41.24	{14., 74.}	87.92	0.282722	Reff	1.25397	0.996433	{0.913689, 1.08632}	20.5378	ar	656.	267.57	{144., 423.}	59.2119
2	R0	{1.73182, 11.3873, 3.}	{1.27112, 4.96652, 0.59}	{{1.06738, 2.19254, 0.}, {1.47488, 12.3431, 3.}}	{26.7882, 66.3079, 83.6667}	inf	37.	13.8764	{3.21156, 23.777}	63.6586	0.532162	newly	33.	26.91	{7., 48.}	48.2727	0.297263	Reff	1.5621	1.17504	{0.991284, 1.36665}	25.0077	ar	653.	329.82	{83., 594.}	50.1746
3	R0	{1.50449, 10.3474, 0.1}	{1.18941, 3.7356, 0.46}	{{1.06738, 2.19254, 0.}, {1.25474, 3.57392, 1.}}	{21.1538, 69.4135, 520.}	inf	32.	14.2537	{3.36698, 28.3416}	57.67	0.48037	newly	23.	32.24	{8., 65.}	76.087	0.301468	Reff	1.30891	1.05549	{0.942245, 1.13045}	19.5814	ar	651.	279.75	{115., 486.}	57.0276
4	R0	{1.63493, 9.1955, 0.1}	{2.17354, 14.8206, 0.35}	{{1.81647, 10.5184, 0.}, {2.56757, 18.9032, 1.}}	{32.9438, 61.1727, 384.}	inf	32.	46.7565	{24.2385, 75.1214}	57.0328	0.183071	newly	23.	26.89	{17., 38.}	33.1739	0.131111	Reff	1.51231	1.97386	{1.67623, 2.31158}	30.5198	ar	663.	820.98	{729., 908.}	24.0181
5	R0	{1.53822, 8.05553, 0.1}	{2.35777, 15.9671, 0.34}	{{2.04669, 12.644, 0.}, {2.93978, 19.9817, 1.}}	{53.2789, 98.2135, 372.}	inf	44.	98.562	{64.6019, 133.894}	124.005	0.334197	newly	31.	51.95	{38., 66.}	67.7097	0.215316	Reff	1.33671	1.91283	{1.68309, 2.24102}	43.0994	ar	621.	856.74	{779., 935.}	37.9614
6	R0	{1.69374, 17.0793, 0.1}	{1.54568, 15.5589, 0.81}	{{1.27063, 10.8657, 0.}, {1.98971, 19.6444, 2.}}	{14.515, 21.0084, 802.}	inf	45.	24.703	{11.0936, 43.1608}	48.2576	0.334098	newly	15.	11.48	{5., 18.}	38.5333	0.223532	Reff	1.54638	1.44136	{1.20155, 1.85185}	13.2523	ar	662.	569.96	{373., 777.}	20.6858
7	R0	{1.42179, 11.8031, 2.}	{1.76373, 13.3417, 0.79}	{{1.52414, 9.52864, 0.}, {1.93087, 18.7084, 2.}}	{24.2884, 23.8805, 60.5}	inf	53.	78.1941	{43.9461, 108.726}	55.5823	0.187468	newly	34.	45.44	{30., 60.}	40.0588	0.143263	Reff	1.16871	1.40206	{1.26824, 1.49223}	20.1249	ar	517.	703.16	{579., 782.}	37.0986
8	R0	{1.46683, 6.95875, 0.1}	{1.9295, 12.5159, 0.95}	{{1.67732, 9.44073, 0.}, {2.315, 19.0355, 2.}}	{31.891, 81.4684, 924.}	inf	33.	54.2119	{25.7308, 93.5017}	71.8025	0.214111	newly	39.	36.24	{21., 50.}	24.4103	0.118883	Reff	1.32308	1.69289	{1.50573, 2.03125}	28.218	ar	653.	754.37	{657., 867.}	16.8714
9	R0	{1.34525, 11.505, 1.}	{1.68151, 15.2075, 1.29}	{{1.5767, 12.7642, 1.}, {1.84468, 19.9256, 2.}}	{26.0002, 37.747, 47.}	inf	25.	46.5932	{26.4498, 67.0112}	91.3667	0.265702	newly	24.	24.29	{15., 35.}	25.625	0.113551	Reff	1.20669	1.46907	{1.37424, 1.60187}	22.6653	ar	532.	663.38	{581., 775.}	29.1842
10	R0	{1.77958, 14.372, 0.1}	{2.17246, 16.8578, 0.06}	{{2.08951, 15.9744, 0.}, {2.22832, 17.2871, 0.}}	{22.0769, 17.9218, 148.}	inf	100.	119.397	{95.344, 147.171}	21.5967	0.0819414	newly	47.	54.89	{42., 69.}	20.617	0.0788553	Reff	1.3685	1.62766	{1.55697, 1.69263}	18.9377	ar	671.	833.73	{777., 873.}	24.2519
11	R0	{1.60334, 19.9548, 1.}	{1.47479, 10.7242, 0.29}	{{1.29991, 8.04006, 0.}, {1.53432, 11.4771, 1.}}	{8.0178, 46.2574, 85.}	inf	56.	36.0391	{22.0054, 48.5666}	35.9121	0.212781	newly	27.	25.84	{16., 33.}	21.3333	0.102503	Reff	1.33398	1.25579	{1.11603, 1.3156}	5.86843	ar	617.	541.36	{376., 638.}	13.5981
12	R0	{1.68623, 7.58585, 0.1}	{2.5504, 14.3541, 0.47}	{{2.0072, 10.1363, 0.}, {2.93488, 18.4249, 1.}}	{51.2485, 89.7499, 476.}	inf	50.	96.2983	{55.8015, 127.923}	93.7383	0.270019	newly	56.	60.56	{46., 75.}	18.7857	0.0775068	Reff	1.4822	2.11546	{1.72999, 2.38262}	42.7247	ar	640.	887.56	{802., 944.}	38.6813
13	R0	{1.82689, 18.0022, 1.}	{2.13272, 17.0198, 0.04}	{{1.87975, 14.1819, 0.}, {2.37462, 19.3966, 0.}}	{17.2742, 11.2208, 96.}	inf	85.	82.9252	{61.3472, 102.217}	16.7112	0.0762073	newly	30.	40.13	{29., 51.}	36.2333	0.128801	Reff	1.51084	1.76593	{1.57285, 1.94426}	17.2522	ar	673.	818.77	{743., 888.}	21.9302
14	R0	{1.75911, 9.16883, 0.1}	{2.41836, 14.4238, 0.36}	{{1.93539, 9.82151, 0.}, {2.88523, 19.0355, 1.}}	{38.7965, 61.4847, 392.}	inf	22.	60.5409	{30.7862, 89.7986}	180.698	0.432058	newly	24.	38.36	{19., 57.}	68.4167	0.220695	Reff	1.63773	2.15154	{1.77096, 2.55562}	32.6439	ar	661.	857.62	{766., 938.}	32.2663
15	R0	{1.46168, 11.3859, 2.}	{1.61004, 11.2047, 1.9}	{{1.39882, 8.26611, 1.}, {1.73739, 13.0272, 2.}}	{11.8482, 15.4191, 13.}	inf	59.	70.9526	{44.8119, 91.5213}	32.0179	0.131884	newly	40.	45.86	{32., 58.}	24.55	0.0988499	Reff	1.10795	1.1989	{1.08548, 1.30582}	9.2455	ar	556.	624.81	{471., 743.}	18.4946
16	R0	{1.53577, 11.7581, 1.}	{2.06624, 12.2754, 0.66}	{{1.44485, 6.00596, 0.}, {2.6365, 19.0355, 2.}}	{37.2346, 30.7896, 62.}	inf	48.	47.493	{13.6992, 82.1918}	37.543	0.195728	newly	31.	34.31	{16., 53.}	36.2258	0.152336	Reff	1.38834	1.86123	{1.33836, 2.28161}	35.998	ar	625.	772.67	{571., 908.}	30.3696
17	R0	{1.70698, 9.72924, 0.1}	{1.69124, 7.77515, 0.68}	{{1.06738, 2.19254, 0.}, {2.86095, 17.1903, 2.}}	{35.69, 65.6389, 700.}	inf	51.	58.5906	{7.84058, 131.3}	85.4157	0.434521	newly	41.	57.31	{20., 88.}	63.9268	0.251995	Reff	1.46288	1.40519	{0.950107, 2.17436}	29.772	ar	675.	528.39	{142., 922.}	45.2933
18	R0	{1.5973, 14.931, 0.1}	{2.17511, 14.8293, 0.89}	{{1.77826, 10.5184, 0.}, {2.76795, 19.5128, 2.}}	{36.6949, 21.888, 864.}	inf	33.	41.6214	{20.3738, 60.5986}	41.0348	0.150927	newly	22.	25.43	{13., 37.}	35.5909	0.138364	Reff	1.48229	1.99541	{1.6354, 2.4278}	35.0723	ar	632.	815.66	{700., 920.}	29.9146
19	R0	{1.87468, 17.004, 1.}	{1.37659, 8.09884, 1.48}	{{1.06738, 2.19254, 0.}, {1.68078, 15.6632, 3.}}	{26.5694, 52.371, 138.}	inf	109.	41.9107	{8.76876, 108.415}	63.1177	0.576277	newly	48.	37.84	{16., 62.}	39.5417	0.233177	Reff	1.37227	1.0941	{0.885983, 1.22875}	20.2706	ar	692.	446.73	{204., 696.}	36.4957
20	R0	{1.84715, 12.531, 0.1}	{2.11921, 17.9825, 0.}	{{1.75668, 13.8428, 0.}, {2.2194, 19.1478, 0.}}	{16.198, 43.5038, 100.}	inf	87.	128.834	{73.5321, 151.371}	53.8273	0.186644	newly	54.	54.42	{39., 66.}	15.	0.0671781	Reff	1.37797	1.48838	{1.34652, 1.56558}	8.89976	ar	661.	817.51	{713., 870.}	24.0045
21	R0	{1.75938, 13.5959, 2.}	{1.72733, 9.50402, 1.39}	{{1.28596, 3.83841, 0.}, {2.24606, 14.662, 3.}}	{19.1721, 37.9398, 62.5}	inf	51.	52.1008	{16.655, 87.8191}	43.7806	0.22899	newly	26.	46.81	{23., 67.}	90.1154	0.269626	Reff	1.52538	1.48514	{1.12074, 1.85006}	16.7785	ar	620.	632.63	{375., 846.}	27.1403
22	R0	{1.63456, 10.7469, 0.1}	{1.82489, 14.278, 0.83}	{{1.42801, 6.00596, 0.}, {1.95665, 16.748, 1.}}	{17.9447, 51.7508, 766.}	inf	101.	58.8018	{35.436, 75.835}	41.7804	0.263484	newly	65.	34.05	{23., 45.}	48.7846	0.307034	Reff	1.27823	1.50117	{1.19671, 1.61719}	20.2772	ar	654.	708.82	{520., 809.}	18.7859
23	R0	{1.62702, 19.1392, 1.}	{1.2429, 4.16818, 0.52}	{{1.06738, 2.19254, 0.}, {1.28596, 3.98513, 2.}}	{24.9951, 78.2218, 98.}	inf	73.	18.7655	{4.56829, 31.64}	74.745	0.726823	newly	31.	33.39	{6., 62.}	51.8387	0.255255	Reff	1.28209	1.04458	{0.904446, 1.09843}	19.8854	ar	600.	316.21	{149., 484.}	49.815
24	R0	{1.70466, 12.1202, 2.}	{1.19343, 3.22667, 0.38}	{{1.06738, 2.19254, 0.}, {1.28596, 3.98513, 1.}}	{29.9903, 73.3777, 82.}	inf	59.	12.8527	{3.93954, 22.6021}	78.2158	0.746762	newly	37.	25.01	{8., 41.}	43.8108	0.298349	Reff	1.25293	0.932093	{0.839454, 0.998925}	25.6066	ar	613.	290.74	{218., 385.}	52.571
25	R0	{1.61735, 11.3169, 0.1}	{1.33156, 6.03006, 0.31}	{{1.08063, 2.5364, 0.}, {1.86254, 19.1994, 1.}}	{22.1815, 69.1295, 348.}	inf	88.	32.5006	{6.38558, 78.9508}	63.9175	0.590286	newly	70.	44.72	{11., 80.}	43.3429	0.315099	Reff	1.2583	1.10458	{0.909136, 1.43771}	17.0595	ar	681.	400.06	{183., 737.}	44.2467
26	R0	{1.56977, 12.3035, 2.}	{2.33002, 15.4585, 0.34}	{{2.11132, 12.644, 0.}, {2.56757, 18.9032, 1.}}	{48.7487, 28.8599, 83.}	inf	74.	103.629	{77.3839, 139.501}	44.6617	0.168206	newly	43.	55.42	{38., 70.}	35.0698	0.138708	Reff	1.30605	1.86352	{1.67559, 2.0695}	42.7859	ar	594.	851.76	{798., 918.}	45.0236
27	R0	{1.46317, 5.8298, 0.1}	{1.15968, 2.64784, 0.03}	{{1.06738, 2.19254, 0.}, {1.28596, 3.98513, 0.}}	{20.8095, 54.5809, 126.}	inf	28.	15.1705	{3.47345, 31.2204}	54.0098	0.424418	newly	37.	40.3	{7., 78.}	59.8378	0.303259	Reff	1.28027	1.02972	{0.939179, 1.14933}	19.7832	ar	586.	270.61	{114., 469.}	53.9676
28	R0	{1.70002, 14.9806, 1.}	{2.42898, 14.4352, 0.55}	{{1.70301, 2.24898, 0.}, {2.96797, 19.6041, 1.}}	{45.7823, 27.871, 53.}	inf	49.	59.6882	{25.5668, 88.2626}	42.6196	0.180767	newly	27.	47.18	{25., 80.}	81.7037	0.209411	Reff	1.54702	2.1783	{1.57506, 2.59755}	43.3461	ar	652.	850.39	{746., 939.}	36.112
29	R0	{1.52729, 16.1014, 3.}	{1.88323, 16.4827, 0.97}	{{1.8189, 14.1819, 0.}, {2.11476, 19.5406, 2.}}	{23.8051, 9.4028, 67.6667}	inf	86.	97.0823	{77.644, 110.737}	18.0677	0.0740708	newly	43.	44.72	{34., 52.}	14.7907	0.0620887	Reff	1.16379	1.41336	{1.35346, 1.57057}	21.658	ar	592.	753.4	{704., 814.}	27.7736
30	R0	{1.72795, 13.5635, 1.}	{1.35336, 5.84823, 0.49}	{{1.06738, 2.19254, 0.}, {1.40191, 6.71734, 2.}}	{22.4816, 56.9394, 85.}	inf	101.	39.369	{15.02, 59.3666}	61.9706	0.484404	newly	52.	46.06	{22., 63.}	26.4231	0.143589	Reff	1.1992	1.01933	{0.837712, 1.07479}	15.6925	ar	668.	462.05	{291., 581.}	32.2859
31	R0	{1.51591, 18.1544, 3.}	{1.6113, 12.8387, 1.92}	{{1.48482, 10.2395, 1.}, {1.66705, 13.8621, 2.}}	{7.96026, 30.2158, 38.6667}	inf	41.	62.2315	{41.5372, 77.4667}	56.3729	0.189489	newly	13.	37.11	{24., 47.}	186.231	0.444151	Reff	1.27185	1.31586	{1.23034, 1.36635}	5.21766	ar	541.	629.1	{512., 707.}	18.536
32	R0	{1.4282, 10.3534, 0.1}	{1.9193, 14.372, 0.43}	{{1.55644, 10.1332, 0.}, {2.24732, 17.1868, 1.}}	{35.5733, 50.0311, 446.}	inf	21.	51.7431	{31.5907, 68.6203}	149.976	0.387103	newly	8.	30.75	{21., 40.}	284.375	0.564979	Reff	1.31109	1.70011	{1.41169, 1.98189}	30.8502	ar	521.	758.09	{556., 858.}	46.8925
33	R0	{1.56693, 11.3972, 0.1}	{2.31752, 13.6386, 0.9}	{{1.87927, 9.63533, 0.}, {2.93488, 18.8445, 1.}}	{48.2687, 30.1161, 832.}	inf	51.	78.0768	{43.6957, 117.058}	61.5884	0.202716	newly	36.	50.06	{35., 69.}	43.2222	0.150539	Reff	1.38203	1.97105	{1.6552, 2.41077}	42.9167	ar	695.	841.33	{748., 931.}	22.177
34	R0	{1.50675, 19.8823, 1.}	{1.25672, 3.69139, 0.25}	{{1.22544, 3.07157, 0.}, {1.28596, 3.98513, 1.}}	{16.594, 81.4338, 93.}	inf	72.	14.9801	{3.76818, 25.6749}	79.1943	0.785835	newly	29.	26.49	{8., 46.}	42.1034	0.254768	Reff	1.0502	0.947531	{0.91587, 0.974455}	9.77651	ar	587.	337.97	{247., 448.}	42.4242
35	R0	{1.72889, 7.76068, 0.1}	{1.67551, 8.91815, 0.86}	{{1.28596, 3.98513, 0.}, {2.35866, 16.9062, 2.}}	{21.9179, 52.5161, 858.}	inf	36.	40.9179	{16.0268, 76.2909}	55.2573	0.225877	newly	45.	38.95	{22., 56.}	31.8	0.15946	Reff	1.55946	1.49351	{1.16159, 2.02346}	19.6089	ar	650.	601.62	{356., 869.}	28.1631
36	R0	{1.62644, 4.12303, 0.1}	{1.23666, 2.80806, 0.}	{{1.06738, 2.19254, 0.}, {1.25474, 3.26445, 0.}}	{24.112, 32.6024, 100.}	inf	30.	29.4025	{6.2176, 51.026}	49.36	0.282589	newly	61.	65.57	{13., 114.}	52.8361	0.282615	Reff	1.31091	0.997182	{0.880521, 1.0382}	23.9321	ar	652.	351.44	{198., 484.}	46.1871
37	R0	{1.70878, 15.8072, 1.}	{1.39508, 8.98986, 0.24}	{{1.38427, 8.35044, 0.}, {1.38427, 8.35044, 0.}}	{18.3584, 43.6812, 108.}	inf	96.	45.0222	{29.2473, 55.9378}	53.1018	0.34193	newly	49.	36.45	{23., 49.}	27.5714	0.154624	Reff	1.21665	1.0643	{1.0411, 1.08506}	12.5222	ar	674.	501.58	{407., 589.}	25.6024
38	R0	{1.58967, 6.80568, 0.1}	{2.18253, 15.4882, 1.71}	{{1.58886, 6.71734, 0.}, {2.48947, 19.255, 2.}}	{39.1494, 129.35, 1630.}	inf	44.	83.205	{42.5025, 110.16}	95.9246	0.286261	newly	51.	44.88	{30., 58.}	18.2745	0.0914551	Reff	1.3846	1.80837	{1.37982, 2.0458}	32.2286	ar	610.	810.61	{594., 900.}	36.3328
39	R0	{1.344, 8.19498, 0.1}	{1.82783, 16.4613, 1.72}	{{1.44485, 6.71734, 1.}, {1.93087, 18.8819, 2.}}	{36.3908, 109.512, 1636.}	inf	37.	80.4458	{37.0184, 103.893}	122.129	0.33627	newly	32.	39.25	{24., 52.}	32.4063	0.118791	Reff	1.1545	1.48702	{1.23984, 1.57788}	29.126	ar	551.	736.65	{582., 809.}	36.4955
40	R0	{1.61509, 7.90825, 0.1}	{1.25146, 3.28286, 0.12}	{{1.06738, 2.19254, 0.}, {1.25474, 3.26445, 0.}}	{27.7456, 67.9165, 210.}	inf	38.	26.7676	{5.05839, 43.9249}	57.2102	0.37598	newly	48.	59.97	{14., 108.}	64.1042	0.282964	Reff	1.39544	1.08799	{0.93671, 1.12009}	25.634	ar	581.	345.51	{128., 499.}	45.5508
41	R0	{1.60017, 5.90605, 0.1}	{2.28453, 15.1152, 0.34}	{{1.81391, 10.5184, 0.}, {2.81891, 18.9032, 1.}}	{42.796, 155.928, 372.}	inf	46.	92.1055	{49.3243, 120.884}	104.053	0.297749	newly	81.	51.09	{35., 65.}	36.9259	0.215349	Reff	1.38735	1.86721	{1.51353, 2.14536}	34.6203	ar	661.	843.3	{716., 922.}	27.7973
42	R0	{1.64436, 15.1337, 1.}	{1.528, 10.5703, 2.3}	{{1.24751, 2.5364, 0.}, {1.79789, 19.6311, 5.}}	{14.2459, 46.2348, 206.}	inf	52.	45.5738	{17.8137, 76.1092}	40.6729	0.229631	newly	26.	39.64	{21., 65.}	63.1538	0.190423	Reff	1.38455	1.29157	{1.08672, 1.48909}	12.113	ar	640.	562.94	{348., 744.}	22.0375
43	R0	{1.48917, 11.917, 0.1}	{1.57829, 12.7138, 0.01}	{{1.59546, 13.1646, 0.}, {1.59546, 13.1646, 0.}}	{7.29149, 12.787, 108.}	inf	67.	60.7159	{45.4815, 73.8583}	14.4701	0.0765944	newly	42.	34.82	{26., 43.}	20.9048	0.106596	Reff	1.16602	1.24515	{1.23242, 1.27285}	7.4682	ar	551.	626.75	{562., 696.}	15.9256
44	R0	{1.65577, 7.89317, 1.}	{1.76383, 12.4322, 1.5}	{{1.36632, 6.00596, 0.}, {2.25962, 18.788, 2.}}	{17.7161, 71.1566, 98.}	inf	24.	37.6196	{18.3372, 58.4555}	69.3368	0.226291	newly	30.	26.44	{16., 38.}	30.6667	0.159936	Reff	1.52331	1.59392	{1.25907, 2.00215}	15.7681	ar	576.	666.17	{452., 863.}	29.2934
45	R0	{1.6909, 12.3275, 0.1}	{1.67851, 11.1141, 1.15}	{{1.38815, 6.71734, 0.}, {2.05052, 16.7932, 2.}}	{16.5945, 36.3313, 1122.}	inf	89.	66.5179	{26.7679, 102.308}	34.4002	0.222163	newly	56.	45.74	{30., 62.}	24.7143	0.137032	Reff	1.29354	1.31385	{1.10808, 1.55316}	13.6345	ar	675.	630.89	{425., 815.}	21.8741
46	R0	{1.62351, 16.0698, 2.}	{1.53184, 11.8494, 1.05}	{{1.38427, 8.26611, 0.}, {1.75663, 14.6725, 2.}}	{11.5452, 26.725, 50.5}	inf	41.	33.9523	{21.8367, 44.9925}	24.575	0.130076	newly	19.	22.89	{15., 33.}	33.6316	0.131519	Reff	1.43681	1.36555	{1.23954, 1.56595}	10.9419	ar	591.	575.35	{444., 716.}	15.6024
47	R0	{1.52372, 4.38599, 0.1}	{2.11664, 11.654, 0.78}	{{1.44485, 5.92488, 0.}, {2.7499, 18.0404, 2.}}	{42.3928, 169.238, 750.}	inf	11.	44.726	{13.5873, 77.7963}	316.611	0.58537	newly	26.	33.73	{15., 49.}	47.9615	0.196023	Reff	1.44449	1.92584	{1.35535, 2.4856}	36.9058	ar	618.	772.25	{537., 921.}	34.6165
48	R0	{1.44306, 11.788, 1.}	{1.56976, 9.65726, 1.04}	{{1.36632, 6.25107, 0.}, {1.81743, 12.8385, 2.}}	{12.5492, 28.4601, 56.}	inf	53.	66.3656	{32.1605, 111.418}	45.3628	0.172842	newly	34.	50.04	{35., 65.}	50.9412	0.175442	Reff	1.12126	1.19352	{1.06111, 1.33059}	9.15124	ar	534.	600.93	{454., 757.}	20.927
49	R0	{1.63624, 11.3893, 1.}	{2.03716, 14.823, 1.91}	{{1.44485, 6.71734, 1.}, {2.45321, 19.391, 3.}}	{28.4384, 43.18, 109.}	inf	71.	66.9701	{27.8804, 94.4067}	29.1652	0.158733	newly	46.	37.82	{26., 49.}	22.	0.116195	Reff	1.39244	1.73407	{1.29907, 2.039}	27.3083	ar	643.	764.95	{506., 884.}	28.4401
50	R0	{1.46113, 11.1322, 1.}	{1.70592, 13.9537, 1.23}	{{1.38815, 8.26611, 0.}, {2.0818, 18.7084, 2.}}	{19.3699, 42.5635, 71.}	inf	34.	37.6798	{13.8885, 58.5612}	43.2757	0.195085	newly	19.	21.12	{11., 31.}	34.5263	0.147794	Reff	1.33547	1.54932	{1.29287, 1.8455}	17.6555	ar	501.	646.19	{430., 806.}	38.2255
